# Integrative Profiling of Phytohormones, Metabolomics, and Transcriptomics Reveals Key Regulators of Cold Tolerance in Cucumber Leaves

**DOI:** 10.1002/fsn3.70027

**Published:** 2025-03-02

**Authors:** Shijun Sun, Shuiyuan Hao, Ye Liu, Xiaoni Gao, Tianlei Mu, Xu Zhang, Yusong Luo, Zhengnan Li

**Affiliations:** ^1^ Hetao College Bayannur Inner Mongolia China; ^2^ Key Laboratory of Urban Agriculture Ministry of Agriculture and Rural Affairs Shanghai China; ^3^ Hetao Green Agricultural Product Safety Production and Warning Control Laboratory Hetao College Bayannur Inner Mongolia China; ^4^ Department of Horticulture Hunan Agricultural University Changsha Hunan China; ^5^ College of Horticulture and Plant Protection, Inner Mongolia Agricultural University Hohhot Inner Mongolia China

**Keywords:** auxin, flavanones, phytohormones, root zone chilling stress, terminal flowering formation

## Abstract

A low‐temperature condition in a root zone is a major abiotic stress that threatens cucumber (*Cucumis sativus L.*) growth and development, yet the molecular mechanism by which the leaf reacts to root zone chilling stress remains largely unknown. In this study, we applied three temperature treatments, including room temperature (20°C–22°C), suboptimal temperature (13°C–15°C), and low temperature (8°C–10°C), to investigate how root zone chilling affects hormone dynamics, metabolomics, and transcriptomics in the leaves of the cucumber variety “Jinyou 35”, the main cultivar in northwest and southwest China. Through integrative physiological and biochemical analysis, auxin emerges as the most significant accumulated hormone, accounting for 88% in room temperature‐treated leaves (RL), 99% in suboptimal temperature‐treated leaves (SL), and 94% in low‐temperature‐treated leaves (LL). Under chilling stress, flavanones were the most abundant metabolite in cucumber leaves, constituting over 50% of total metabolites, while phenolic acids showed a marked decrease. Several differentially expressed transcription factors (DETFs), such as *LOB* (*CsaV3_3G020650*), *MYB* (*CsaV3_3G043510*), and *bHLH* (*CsaV3_2G005070* and *CsaV3_4G029740*), were upregulated in SL and LL, potentially enhancing cucumber's defense against chilling injury. Additionally, terminal flower formation was observed under suboptimal and low‐temperature conditions, with *CsFT* expression in SL and LL lower than in RL, and a significant negative correlation observed between *CsFT* and *CsNAC6*. These findings deepen our understanding of cucumber's resilience mechanisms to root zone chilling stress, shedding light on its cold tolerance strategies.

## Graphical abstract

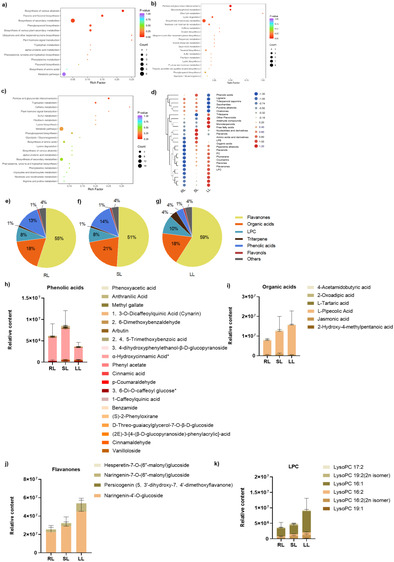

## 1 Introduction

Plants face numerous environmental threats during the whole growth and development stages. Among these, low temperature is a critical abiotic stressor that affects plant growth, development, yield, and quality (Peng et al. 2014; Xu et al. 2023; Zhou et al. 2021). Low‐temperature stress impacts plants from seed germination to flowering and fruiting stages, and chilling (occurring above 0°C) and freezing injury (occurring below 0°C) are two major categories (Chen et al. 2020; Jan et al. 2009). Chilling injury disrupts the cell membrane structure and function, increasing permeability of the cellular membrane and ion transmembrane fluxes, which breaks the ion homeostasis across the cell membrane (Bai et al. 2021). This stress also influences enzymatic activity, leading to imbalances in metabolism (Zhao et al. 2021). In contrast, freezing injury can cause intracellular and extracellular ice formation, protein degradation, and harm to biofilm systems (Xu et al. 2023). Severe freezing injury may result in plant death (Saadati et al. 2021). Therefore, while chilling and freezing injuries are both forms of low‐temperature stress, they differ in mechanisms and outcomes (Chen et al. 2014; Gong et al. 2020). Cucumber, a plant that undergoes both vegetative and reproductive growth simultaneously, has two main inflorescence types: indeterminate and determinate (Ratcliffe et al. 1998; Wen et al. 2019). In indeterminate plants, the main stem grows continuously, producing flowers along its sides, while in determinate plants, the main stem stops growing as the shoot apical meristem culminates in flowering. Growth and terminal flower formation are serious problems caused by chilling injuries in cucumbers. Cucumbers, originating from the southern foothills of the Himalayas, are now widely cultivated and economically valuable. According to FAO, China harvested 1,311,461 ha of cucumbers in 2020, yielding 58,947.4 kg·ha^−1^ (https://www.fao.org/zh). Cucumbers thrive in high temperatures, particularly during the seedling stage, making them sensitive to low temperatures. Different varieties exhibit varying resistance to cold (Li et al. 2022). In northern China, cucumbers are grown in early spring and winter, but low temperatures during early planting can hinder growth, delay fruiting, and cause economic losses (Li et al. 2022). Addressing chilling injury is particularly urgent for crops like cucumber grown annually in controlled environments.

Phytohormones, like auxin, abscisic acid (ABA), brassinosteroid (BR), gibberellin (GA), and jasmonate (JA) are known to play vital roles in adaptation to cold stress (Ding et al. 2015; Eremina et al. 2016; Hu et al. 2013; Lantzouni et al. 2019; Li et al. 2017). Studies show ABA levels increase in *Carpobrotus edulis* during cold stress (Fenollosa et al. 2018), and exogenous ABA application enhances freezing tolerance in grapevines (Wang, Blakeslee, et al. 2020). Auxin, crucial for organogenesis, is affected by cold stress, which disrupts auxin transport and inhibits its asymmetric distribution (Rahman 2013; Shibasaki et al. 2009). JA is an upstream component regulating the CBF‐expression (ICE)‐CBF/DREB1 pathway, playing key roles in mediating cold tolerance in plant (Hu et al. 2013).

Flavonoids serve various functions in plants, from flower and fruit pigmentation to regulating auxin transport and defending against pathogens and UV light (Nakabayashi et al. 2014; Winkel‐Shirley 2002). They also accumulate in response to stresses, for instance, drought, frosty, and intense light, mediating stress tolerance (Nakabayashi et al. 2014; Winkel‐Shirley 2002). Low temperatures, in particular, significantly increase flavonoid content, the activity of flavonoid biosynthesis enzymes (Korn et al. 2008; Schulz et al. 2015), and the induction of cold resistant genes (Crifò et al. 2011; Kaplan et al. 2007), often resulting in purple leaves due to anthocyanin accumulation, a common indicator of plant stress. Cold stress activates several cold resistant pathways in plants, notably the transcription of *ICE1‐C‐repeat‐binding factors‐cold‐regulated genes* (*ICE1‐CBF‐COR*) gene cascade (Barrero‐Gil and Salinas 2018). This pathway quickly induces CBF/DREB complexes that bind to COR gene promoters, initiating transcription (Shi et al. 2015; Yang 2022). These signaling pathways also upregulate multigene families of transcription factors, including *Myeloblastosis* (*MYB*), *WRKY*, *NAM* (*No Apical Meristem*), *ATAF1/2*, *and CUC2* (*Cup‐Shaped Cotyledon*) (*NAC*), *Basic Leucine Zipper* (*bZIP*), and *APETALA2*/*Ethylene‐Responsive Element Binding Factor* (*AP2/ERF*), which are crucial for stress response (Kidokoro et al. 2021; Song et al. 2021; Wang, Hao, et al. 2020). Chilling injury affects plant transition from vegetative to reproductive growth by influencing multiple genetic pathways. For instance, *TERMINAL FLOWER 1* (*TFL1*) and *FLOWERING LOCUS T* (*FT*) belonging to the phosphatidylethanolamine‐binding protein (PEBP) family are two primary regulators for floral transition in *Arabidopsis* (Baumann et al. 2015; Ratcliffe et al. 1998; Shannon and Meeks‐Wagner 1991; Wickland and Hanzawa 2015).

Nowadays, transcriptomic and metabolomic analyses are of great essence for dissecting the underlying molecular components in plant response to low‐temperature stresses (Bahrman et al. 2019; Xu et al. 2023). This study combines transcriptomics and metabolomics to investigate the cold acclimation in cucumber leaves under three different root zone temperatures treatments: optimal (18°C–20°C), suboptimal (13°C–15°C), and low temperature (8°C–10°C). Using the cucumber cultivar “Jinyou 35,” we successfully examined hormone dynamics and cold tolerance‐related genes and metabolites in leaves upon chilling stress.

## 2 Materials and Methods

### 2.1 Plant Materials and Growth Conditions

The cultivar “Jinyou 35” was selected in our study for its superior cold resilience and extensive cultivation area in China, which reached over 70% by 2010 (Sun et al. 2016, 2018, 2017, 2024). As of now, “Jinyou 35” remains the predominant cucumber cultivar in Inner Mongolia (Sun et al. 2016, 2018, 2017). All experiments in this study were conducted in the Crop Cultivation Laboratory at Hetao College, located in Bayannaoer and Inner Mongolia Province (40°34′–41°17′ N; 107°6′–107°44′ E). Three distinct conditions were applied: an optimal range of 20°C–22°C, which served as the control (CK), a suboptimal range of 13°C–15°C, and a low‐temperature range of 8°C–10°C. The humidity is 70%–80%. The range of light intensity is 8000–10,000 lx, and duration is 8–10 h. The substrate used for plant cultivation consists of peat, vermiculite, and perlite in a volumetric ratio of 6:3:1. Seedlings are cultivated in this substrate with 12 cm space in line and 18 cm space in row in mid‐July 2022.

The temperature was regulated and monitored by a controller positioned 15–18 cm beneath the substrate surface, while the probe was embedded 8 cm under the substrate surface. The temperature was adjusted during 22:00–6:00 (the next day). The three temperature conditions were employed as experimental treatments. Each temperature group consisted of 60 seedlings. The leaves positioned beneath the flowers of the: “Jinyou 35” cultivar were sampled. 20 days post‐transplantation, the frozen leaf samples selected were delivered to Wuhan Metware Biotechnology Co. Ltd. for analysis of phytohormone levels, as well as transcriptomic and metabolomic assessments. Leaf samples were designated as RL (room temperature), SL (suboptimal temperature), and LL (low temperature). Each test was replicated three times to ensure reliability.

Under suboptimal and low‐temperature conditions in the root zone, the development of terminal flowers was observed in the cucumber plants.

### 2.2 Analysis of Endogenous Hormone

The frozen leaf samples were grounded into a fine powder and kept in −80°C for analysis. Approximately 50 mg leaf samples were weighed into 2 mL microtubes and subsequently dissolved in a 1 mL methanol/water/formic acid mixture (15:4:1; V/V/V). 10 μL of an internal standard solution (100 ng/mL) was added to the extract for quantification. The mixture was vortexed for 10 min and then centrifuged at 16099.2 xg for 5 min at 4°C. The supernatant was then transferred to new microtubes, evaporated to dryness, dissolved in 100 μL 80% methanol (V/V), and filtered through a 0.22 μm membrane filter for subsequent LC–MS/MS analysis (Floková et al. 2014; Li et al. 2016).

The UPLC‐ESI‐MS/MS system was used (UPLC; ExionLC AD; MS; Applied Biosystems 6500 Triple Quadrupole). Parameters are detailed in Table S1. High‐grade CH_3_CH and CH_3_OH for HPLC were procured from Merck (Darmstadt, Germany). Milli‐Q H_2_O is sourced from Millipore (Bradford, USA). CH_3_COOH and HCOOH were purchased from Sigma‐Aldrich (St. Louis, Missouri, USA). The 1 mg/mL stock solutions were prepared with CH_3_OH and stored at −20°C. For analysis, stock solutions were diluted in CH_3_OH to create the required working solutions.

The QTRAP 6500+ LC–MS/MS system is equipped with an ESI Turbo Ion Spray interface and the Analyst 1.6.3 software. The settings used for ESI are as follows: ion source, ESI +/−, source temperature 550°C, ion spray voltage 5500 V (positive) and −4500 V (negative), curtain gas (CUR) 35 psi. The analysis of plant hormones was conducted using multiple reaction monitoring (MRM). Analyst 1.6.3 software acquires the data, and the Multiquant 3.0.3 software quantifies all metabolites. The optimized settings used for mass spectrometry include the declustering potentials (DPs) and collision energies (CEs). MRM transitions were monitored during the metabolites eluting (Cui et al. 2015; Pan et al. 2010; Šimura et al. 2018).

### 2.3 Metabolite Profiling and Data Analyses

The samples dried in vacuum freeze‐dryer (Scientz‐100F) are pulverized with mixer mill (MM 400; Retsch) employing zirconia beads (1.5 min/30 Hz). 50 mg of lyophilized powder was dissolved in 1.2 mL of 70% CH_3_OH and vortexed (30 s/30 min in 6 cycles). Then, the solution was centrifuged (12000 rpm; 3 min), and the supernatant was filtered (SCAA‐104; 0.22 μm pore size; ANPEL; Shanghai; China) prior to performing UPLC–MS/MS analysis.

The meticulous analysis was performed using UPLC‐ESI‐MS/MS system. The UPLC was equipped with an Agilent SB‐C18 column (1.8 μm; 2.1 × 100 mm). The mobile phase comprised of solvent A (pure water fortified with 0.1% formic acid) and solvent B (acetonitrile with 0.1% formic acid). The gradient program initiated with 95% A and 5% B, transitioning linearly to =5% A and 95% B over 9 min, followed by maintenance of this composition for 1 min. The composition then returned to 95% A and 5% B over 1.1 min and stabilized for 2.9 min. The flow rate was set at 0.35 mL/min, and the column oven temperature was maintained at 40°C, with an injection volume of at 4 μL. The effluent was directed to an ESI‐triple quadrupole‐linear ion trap‐MS (QTRAP)‐MS system for further analysis.

Operational parameters for the ESI source included a source temperature of 550°C and an ion spray voltage of 5500 V (positive polarity) and −4500 V (negative polarity), with ion source gases I (GSI), II (GSII), and CUR set at 50, 60, and 25 psi, respectively. High collision‐activated dissociation was utilized during the MRM experiments, with QQQ scans acquired using nitrogen as the collision gas. The values of DPs and CEs for individual MRM transitions were precisely optimized, with a dedicated set of transitions monitored for each metabolite eluting during the analysis.

For two‐group analysis, differential metabolites were identified based on VIP scores (VIP ≥ 1) and absolute value of Log2FC (|Log_2_FC| ≥ 1.0). VIP values were derived from OPLS‐DA results, including score and permutation plots generated using the R package MetaboAnalystR. Prior to OPLS‐DA analysis, data underwent log transformation (log_2_) and mean centering, and a permutation test with 200 iterations was performed to minimize over‐fitting.

Identified metabolites were annotated using the Kyoto Encyclopedia of Genes and Genomes (KEGG) Compound database (http://www.kegg.jp/kegg/compound/). Subsequently, these annotated metabolites were mapped to the KEGG Pathway database (http://www.kegg.jp/kegg/pathway.html). Pathways related to significantly regulated metabolites were then subjected to metabolite sets enrichment analysis (MSEA), with significance evaluated using hypergeometric test p‐values.

### 2.4 RNA‐Seq and Transcriptomic Data Analysis

The leaf of “Jinyou 35” was frozen in liquid N_2_ and stored in a fridge (−80°C). The Plant Total RNA Extraction Kit (TIANGEN, China) for isolating RNA from the leaves was purchased. RNA (OD A260/A280 [1.9–2.1]; A260/A230 [> 2.0]) was selected for the cDNA synthesis. RNA‐seq was subsequently performed by Mateware using Illumina HiSeq4000 (Illumina, San Diego, USA). For raw data pre‐processing, Fastp was utilized to remove adapters and low‐quality sequences. Clean reads alignment to the reference genome (ChineseLong_genome_v3.fa.gz) was performed with HISAT2. Statistics of mapped reads counts, transcript lengths, and FPKM indicate the transcription levels. The Pearson correlation coefficient and principal component analysis were employed to assess the correlation and reproducibility among samples. Differentially expressed genes (DEGs) were identified by DESeq2 with thresholds set at |log_2_‐fold Change| ≥ 1 and FDR < 0.05. Each leaf sample was subjected to three biological replicates.

Identified non‐redundant transcript sequences classified as genes underwent further analysis through Gene Ontology (GO) annotation to discern significant GO terms among DEGs. Additionally, the KEGG database was employed to pinpoint significantly enriched metabolic pathways.

Reverse transcription quantitative polymerase chain reaction (RT‐qPCR) validation of key genes from RNA‐seq data was conducted. The cucumber leaf samples of the “Jinyou 35” were stored at −80°C. The cDNA was synthesized using TAKARA PrimeScript RTMaster Mix (Perfect Real Time). qPCR was conducted on a QTOWER Real‐time fluorescence quantitative PCR instrument (ANALYTIKJENA, Germany) following the manufacturer's instructions using Talent qPCR PreMix (SYBR Green). The actin gene of cucumber was selected as the internal control for normalization. Each reaction was replicated three times, with a total reaction volume (20 μL). The following settings were used for qPCR: denaturation (96°C for 10 min), followed by 40 cycles (95°C, 15 s, 58°C, 20 s, 72°C, 30 s), culminating in last step (72°C, 10 min). Melting curve analysis shows the specificity of amplification. Gene relative expression was calculated by 2^−ΔΔ*C*^_T_ method. The primers are listed in Table S2. All samples were evaluated individually with three biological and technical replicates.

### 2.5 Statistical Analyses

Each experiment was conducted three times, and the values are shown as mean ± standard deviation (SD). SPSS Statistics 26.0 software was applied to assess the significant differences (*p* < 0.05) using one‐way analysis of variance (ANOVA), followed by Duncan's multiple range test. The comparison groups for hormone, metabolite, and RNA‐seq data were designated as RL_versus_SL, RL_versus_LL, and SL_versus_LL, respectively.

## 3 Results

### 3.1 Endogenous Phytohormone Profiling of RL, SL, and LL

**FIGURE 1:**
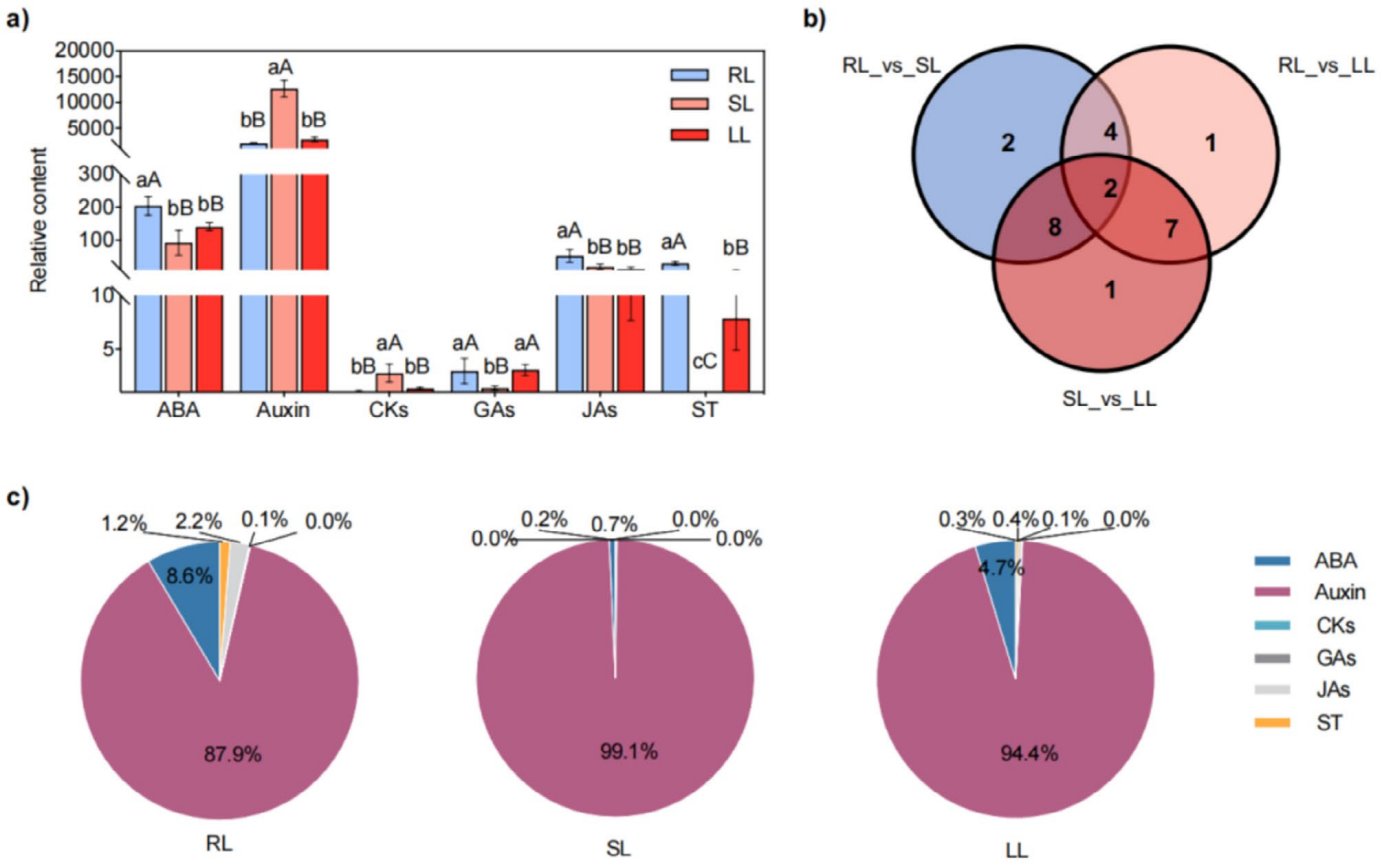
RL, SL, and LL exhibit distinct phytohormone profiling. (a) Relative content of ABA, auxin, CKs, GAs, JAs, and ST in RL, SL, and LL. (b) Number of differentially accumulated endogenous hormones in RL_versus_SL, RL_versus_LL, and SL_versus_LL. (c) Proportions of ABA, auxin, CKs, GAs, JAs, and ST in RL, SL, and LL. Letters in (a) represent the significant differences (lowercase, 0.05 > *p* ≥ 0.01); uppercase, (0.01 > *p* ≥ 0.001).

In this study, the hormone profiling was conducted with leaves beneath the terminal flower of the cucumber cultivar “Jinyou 35”, whose roots are treated with room temperature, suboptimal temperature, and low temperature. These leaves were designated as RL, SL, and LL, respectively. A total of 25 plant hormones, belonging to six phytohormone categories, including ABA, auxins, cytokinins (CKs), GAs, JAs, and strigolactone (ST), have been quantified in these leaves using the HPLC‐ESI‐MS system. As depicted in Figure 1, both SL and LL exhibited significantly lower ABA, JA, and SL contents than RL, while ST displayed significantly higher auxin and CKs contents than RL and LL, but lower GAs and ST contents than RL and LL. The detailed comparison results of RL versus SL, RL versus LL, and SL versus LL are listed in Table S3, in which all 25 examined phytohormones can be categorized as differentially accumulated endogenous hormones. The Venn diagram in Figure 1b clearly exhibits the quantities of differentially accumulated endogenous hormones of RL versus SL, RL versus LL, and SL versus LL are 16, 14, and 18, respectively. Notably, two differentially accumulated endogenous hormones (2MeSiP and ST) are shared by all three comparison groups, suggesting their potential in regulating cucumber's response to low temperatures. Furthermore, the proportions of these six phytohormones as a whole were calculated, and auxin is the most dominant hormone occupying 87.9% in RL, 99.1% in SL, and 94.4% LL, as shown in Figure 1c. The proportions of ABA and GAs undergo dramatic decrease in SL compared to RL. However, their proportions are partially restored in LL, indicating the influence of cold treatment at the root zone on the dynamics of leaf phytohormones. In short, temperature reduction induces notable changes in leaf hormone levels, with ABA, GA, and ST exhibiting a decrease in SL than RL and an increase in auxin content in SL than RL. As the most abundant hormone in cucumber leaves, auxin is of great significance in regulating leaf cold resistance.

### 3.2 Differentially Expressed Metabolites of RL, SL, and LL

To better understand the metabolite changes in cucumber leaves upon low‐temperature treatment in root, LC–MS/MS was used to perform untargeted metabolomics analysis. DEMs were then analyzed in three groups, including RL_versus_SL, RL_versus_LL, and SL_versus_LL. Orthogonal partial least squares discriminant analysis (OPLS‐DA) is primarily used for identifying key DEMs between temperature treatments (Figure S1d–f). Subsequently, MRM was applied to validate and quantify the DEMs with high accuracy and sensitivity, followed by correlation analysis to dissect the interrelationship between low temperature and DEMs (Figure S1a–c). Out of 103 metabolites screened for each comparison group, 42 DEMs presented in RL_versus_SL (32 up‐ and 10 downregulated), 64 DEMs found in the RL_versus_LL comparison (38 upregulated and 26 downregulated), and 61 DEMs found in the SL_versus_LL comparison (25 upregulated and 36 downregulated) (Figure S1g–i). Venn diagram analysis further showed 14 DEMs shared by RL_versus_SL and RL_versus_LL. The most dominant DEMs are categorized as flavonoids, phenolic acids, amino acids, and their derivatives. Two DEMs were common to all three groups. One is a flavanone, and the other is a pyridine alkaloid. Flavonoids are capable of scavenging ROS to increase the resilience of plant against biotic stress, suggesting that plants increase suboptimal and low‐temperature tolerance by accumulating antioxidant enzymes.

**FIGURE 2:**
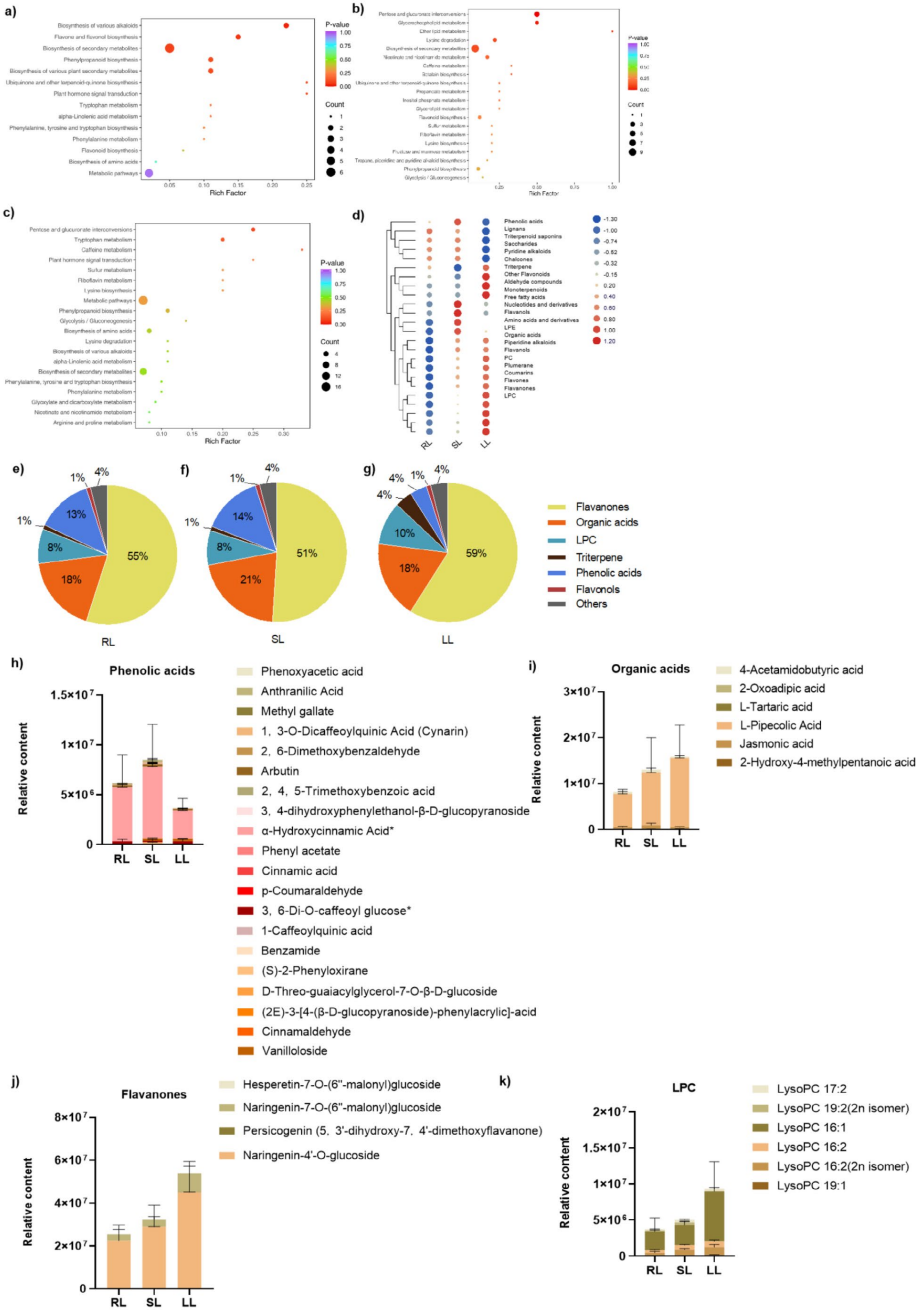
DEM analysis of RL_versus_SL, RL_versus_LL, and SL_versus_LL. The KEGG enrichment of DEMs of RL_versus_SL (a), RL_versus_LL (b), and SL_versus_LL (c). (d) Cluster heatmap of 24 selected DEMs. (e–g) Pie charts show the proportions of most abundant metabolites classified as Class II. Stacked bar chart of composition of phenolic acids (h), organic acids (i), flavanones (j), and LPC (k).

**FIGURE 3:**
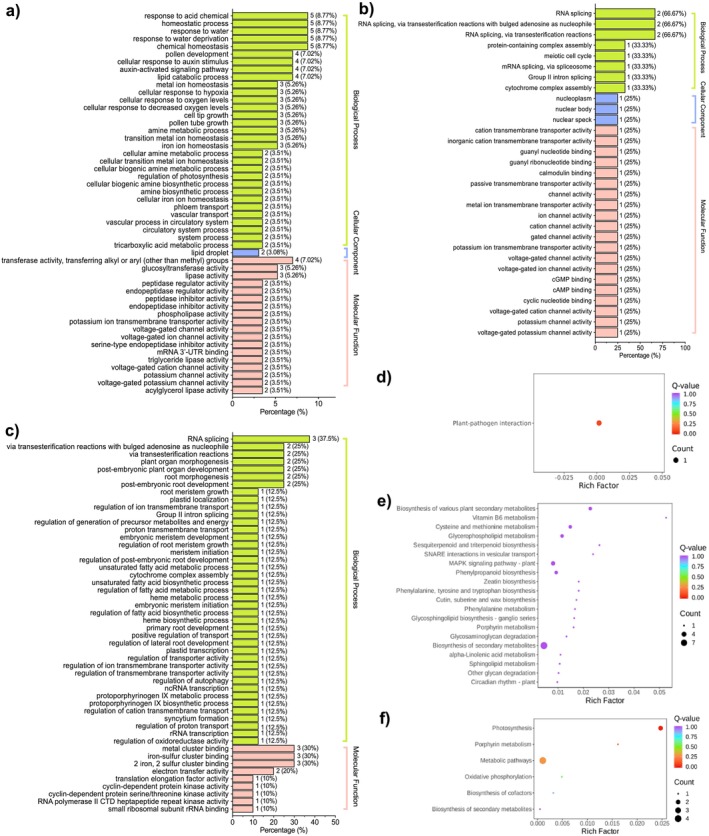
DEG analysis of RNA‐seq. Go enrichment of DEGs in RL_versus_LL (a), RL_versus_SL (b), and SL_versus_LL (c). The KEGG enrichment of DEGs in RL_versus_SL (d), RL_versus_LL (e), and SL_versus_LL (f).

As shown in Figure 2a, 42 DEMs of RL_versus_SL were significantly enriched in pathways, such as “biosynthesis of various alkaloids (ko00996)”, “flavones and flavonols (ko00944)”, and “secondary metabolites (ko01110)” based on KEGG annotation. These results indicate that cucumber leaves accommodate to suboptimal temperature stress by increasing the abundance of alkaloids and flavones. Likewise, 64 DEMs of RL_versus_LL were significantly enriched in 20 pathways, such as “pentose and glucuronate interconversions (ko00040)”, “glycerophospholipid metabolism”, and “ether lipid metabolism” (Figure 3b). By contrast, 61 DEMs of SL_versus_LL were enriched in 20 pathways, such as “pentose and glucuronate interconversions”, “tryptophan metabolism”, and “caffeine metabolism” (Figure 3c). Intriguingly, the DEMs categorized as “pentose and glucuronate interconversions” were shared by SL_versus_LL and RL_versus_LL and downregulated (5/6), suggesting that cucumber leaves responded to chilling injury by reducing the activity of this pathway.

As shown in Figure 2d a cluster heatmap exhibits 24 selected DEMs in RL, SL, and LL. Among the flavones, flavanone, lysolecithin (LPC), coumarins, PC (phosphatidyl choline), flavonols, piperidine alkaloids, organic acid, LPE (Lysophosphatidyl ethanolamine), aldehyde compounds, other flavonoids, and triterpene were significantly more accumulated than the other metabolites in LL than RL and SL and display an increasing accumulation trend with decreasing temperature. By contrast, phenolic acids, lignans, triterpene saponin, saccharides, and pyridine alkaloids are downregulated in LL than RL and SL, showing their negative correlation with chilling stress in plants (Figure 2d). Among these metabolites, flavanones are the most abundant metabolite, accounting for more than 50% of all metabolites with 55% in RL, 51% in SL, and 59% in LL (Figure 2e–g). Naringenin‐4'‐O‐glucoside (HJN087) is the major flavanones accumulated in cucumber leaves, of which the relative content and detailed information are shown in Table S4. L‐pipecolic acid (MWS0811) was the main organic acid, and LysoPC 16:1 (pmp001270) was the main component of LPC (Figure 2i,k and Table S4). The organic acid accounts for 18% in RL, 21% in SL, and 18% in LL, showing a slightly increasing trend in content upon temperature decrease. The proportions of LPC in RL, SL, and LL are 8%, 8%, and 10%, respectively (Figure 2e–g). Intriguingly, the proportion of phenolic acids is 13% in RL and 14% in SL, while it sharply decreases to 4% in LL (Figure 2h).

### 3.3 RNA‐Seq Analysis of RL, SL, and LL

To further investigate the DEGs in cucumber seedlings affect by chilling stress, transcriptomic analyses were conducted using the leaves of cucumber seedlings. Nine cDNA libraries were prepared for sequencing. The quality metrics for each cDNA library were high, with Q20 and Q30 values exceeding 97% and 92%, respectively. The base error rate was 0.03%, and the average G/C content was 42.76%. The average number of bases was 6.8 Gb (Table S5). More than 95% of the clean reads mapped to the cucumber reference genome. Additionally, the square of the correlation coefficient (*R* ^2^) based on the FPKM values was consistently larger than 0.95 (Figure S2a), indicating high reproducibility among biological replicates.

DEG analysis exposed that 94 DEGs were identified (58 upregulated and 36 downregulated) in the RL_versus_LL comparison. The SL_versus_LL comparison yielded 20 DEGs (6 upregulated and 14 downregulated), while the RL_versus_SL comparison had the fewest DEGs, with only 5 (3 upregulated and 2 downregulated) (Figure S2b). Venn diagram analysis showed that there was only one shared DEG between the RL_versus_SL and RL_versus_LL groups and two common DEGs between RL_versus_SL and SL_versus_LL groups (Figure S2c). Remarkably, there were 11 shared DEGs identified between the RL_versus_LL and SL_versus_LL groups. Intriguingly, no shared DEGs were identified across all three comparisons, suggesting that the cucumber response to suboptimal and low temperatures involves distinct sets of genes.

Furthermore, GO enrichment analysis of the DEGs in each comparison group was conducted. In RL_versus_SL and SL_versus_LL comparisons, DEGs were significantly enriched in “RNA splicing” (GO:0008380), “RNA splicing, transesterification reactions” (GO:0000375), and “RNA splicing via transesterification reactions with bulged adenosine as a nucleophile” (GO:0000377) (Figure 3a,c). In contrast, DEGs in the RL_versus_LL comparison were enriched in processes related to “response to acidic chemicals” (GO:0001101), “chemical homeostasis” (GO:0048878), and “homeostatic processes” (GO:0042592), among others (Figure 3b).

KEGG pathway enrichment analysis revealed that one DEG (*CsaV3_3G047190*) in the RL_versus_SL comparison was enriched in the “plant–pathogen interaction” (ko04626) (Figure 3d). In the RL_versus_LL comparison, 63 DEGs were enriched across 34 pathways (Figure 3e), with significant enrichment in the “biosynthesis of various plant secondary metabolites” (ko00999), “Vitamin B6 metabolism” (ko00750), and “amino acid metabolism” (ko00270). These pathways suggest that cucumber leaves respond to chilling stress using these fundamental metabolic pathways. In the SL versus LL comparison, 10 downregulated DEGs were enriched in six pathways (Figure 3f), with significant involvement in “photosynthesis” (ko00195), “porphyrin metabolism” (ko00860), “metabolic pathways” (ko01100), and “oxidative phosphorylation” (ko00190). These results indicate that low‐temperature stress, as opposed to suboptimal temperatures, disrupts photosynthetic pathways, potentially contributing to the formation of terminal flowers in cucumbers under low‐temperature conditions.

### 3.4 Transcription Factors Related to Cold Stress

**FIGURE 4:**
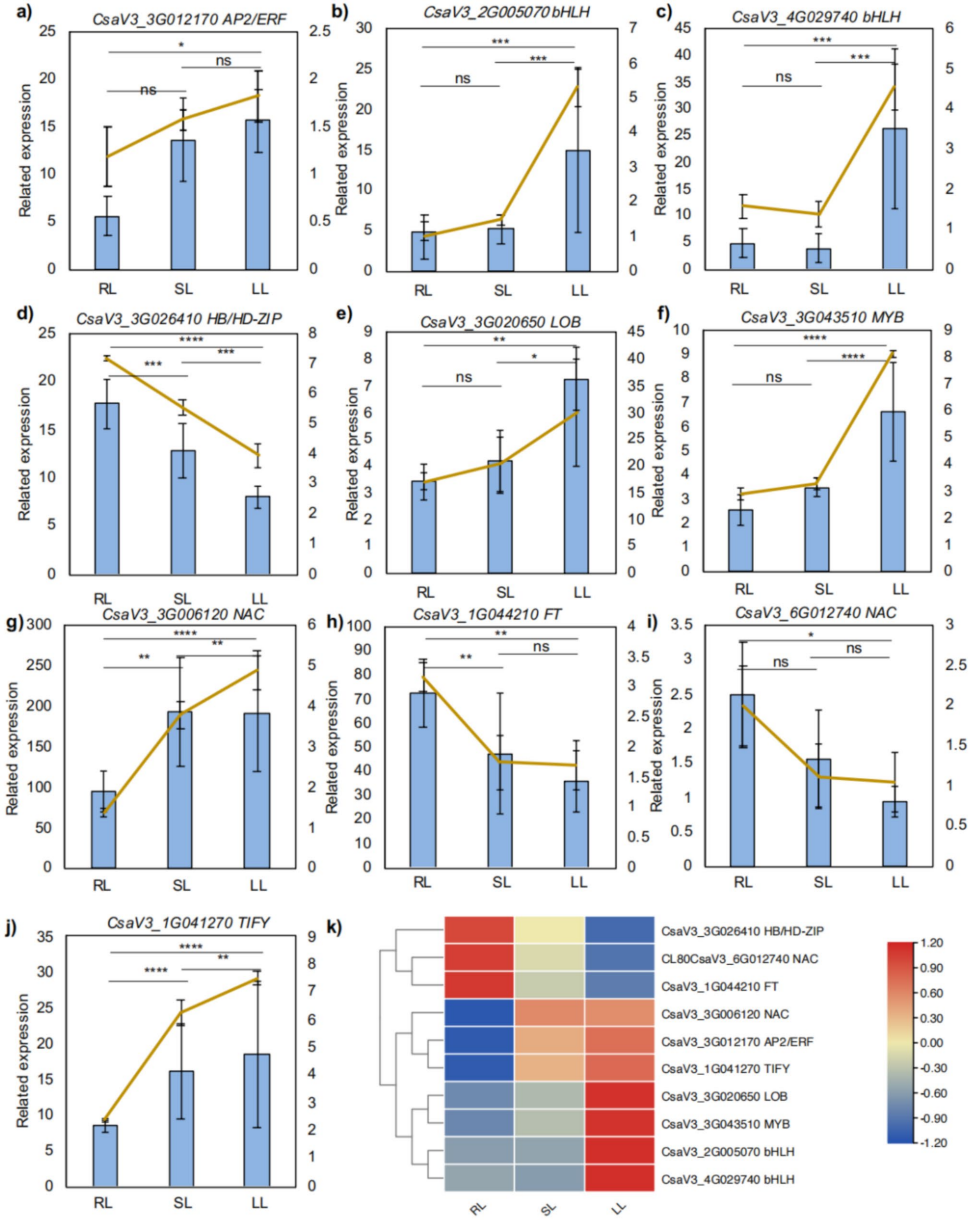
Heat map and RT‐qPCR verification of DETFs. The blue bar represents the transcriptome data, and the orange line represents the RT‐qPCR data (a–j). Heatmap of transcriptomic results of 10 DETFs (k). Results of RT‐qPCR were first normalized according to the constitutively expressed protein actin gene. The error bars in (a–j) graphs depict mean ± SD, *n* = 3. *p* value of RT‐qPCR results (orange lines) was determined by one‐way ANOVA analysis; NS, not significant; ****p value < 0.0001, ***p value < 0.001; **p value < 0.01; *p value < 0.05.

The differentially expressed transcription factors (DETFs) and the *FT* (*CsaV3_1G044210*) gene are presented in Figure 4. Most DETFs were upregulated in SL and LL, including *NAC* (*CsaV3_3G006120*), *AP2/ERF* (*CsaV3_3G012170*), *TIFY* (*CsaV3_1G041270*), *LOB* (*CsaV3_3G020650*), *MYB* (*CsaV3_3G043510*), and *bHLH* (*CsaV3_2G005070* and *CsaV3_4G029740*). These DETFs are positively correlated to the cucumber's response to chilling stress. Notably, the expression of bHLH family genes was significantly higher under low‐temperature conditions compared to both RL and SL.

Additionally, *CsTI10A*, belonging to the TIFY family, is implicated in flowering regulation and stress response through the JA signaling pathway. In contrast, *FT* is known to negatively regulate flowering in cucumber plants (Pan et al. 2017). The expression patterns of DETFs such as NAC (*CsaV3_6G012740*, *CsJA2L*), *C2H2* (*CsaV3_5G012400*), and *HB* (*CsaV3_3G026410*) were found to be similar to those of *CsFT*. Among these, CsNAC6 in the NAC family showed the strongest correlation with *CsFT*, suggesting that *CsNAC6* may negatively regulate flowering and low‐temperature stress in response to *CsFT*. The RT‐qPCR results were consistent with the RNA‐seq results, suggesting the reliability of the sequencing results (Figure 4).

### 3.5 Comparative Analysis of DEGs and DEMs of RL, SL, and LL

A combined analysis of multi‐omics contributes to the prediction of metabolite changes at the transcriptional level and helps verify gene expression results through metabolic profiling. The interaction between transcriptional and metabolic pathways is crucial for the metabolic mechanisms in various biological systems in plants (Cavill et al. 2016). To explore this further, the DEGs and DEMs identified in leaves under terminal flowers in low‐temperature conditions were mapped to KEGG pathway database for comparison. No co‐enriched pathways were found in the RL versus SL comparison group. However, in the 3 comparison groups, 11 and 3 pathways were co‐enriched by both DEGs and DEMs.

In the RL_versus_LL comparison, DEGs and DEMs were significantly enriched in the “biosynthesis of various plant secondary metabolites”, “cysteine and methionine metabolism” (ko00270), “glycerophospholipid metabolism” (ko00564), “phenylpropanoid biosynthesis” (ko00940), and other pathways (Figure S3a). For the SL_versus_LL comparison, significant enrichment was observed in metabolic pathways, “cofactor biosynthesis” (ko01110) and “secondary metabolite” (Figure S3b). These shared pathways represent the combined multi‐omics foundations for cucumber leaves' resistance to low temperatures, which may contribute to the formation of terminal flowers under stress conditions.

**FIGURE 5:**
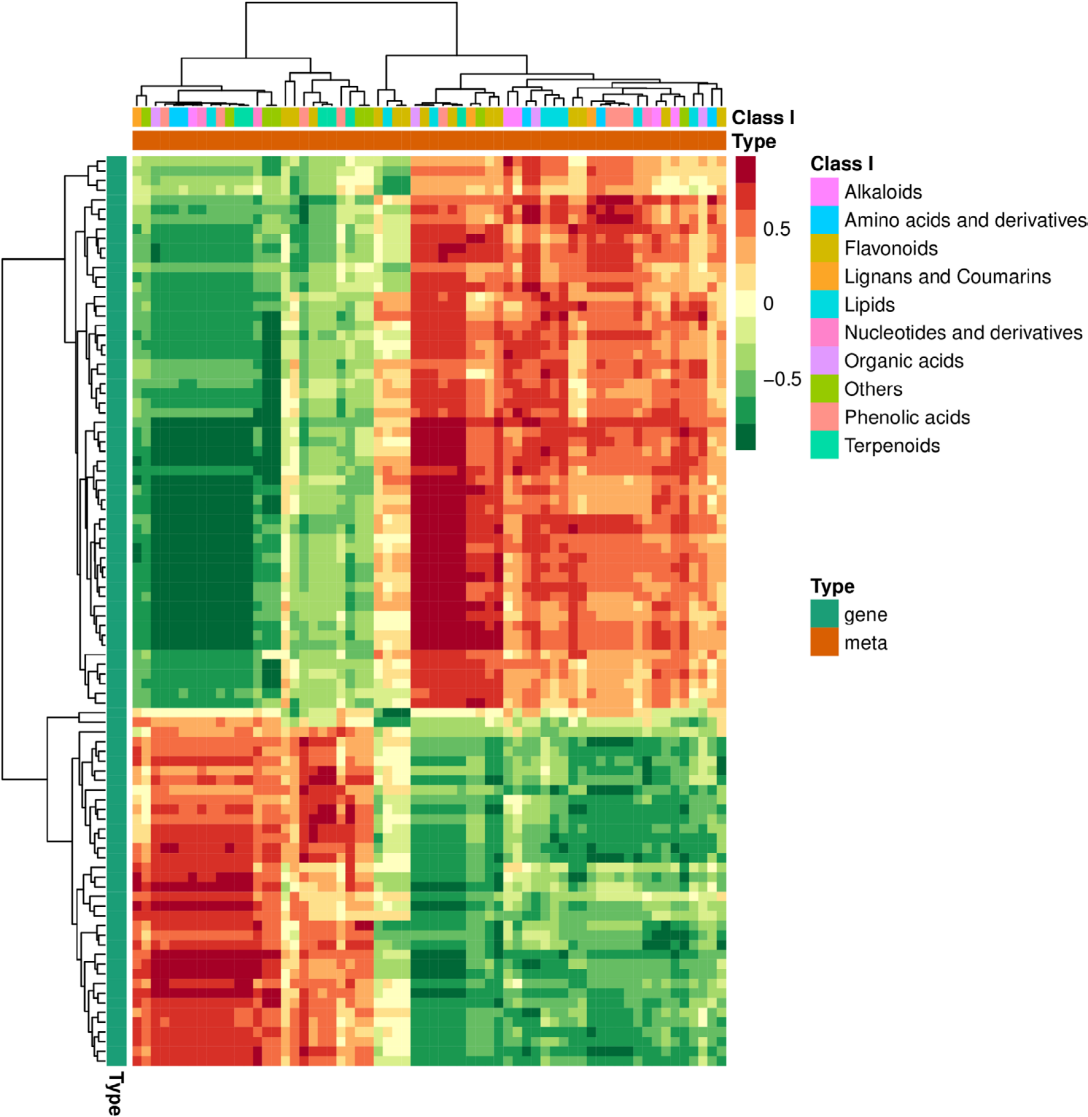
Heatmap of correlationship between metabolites and transcriptomes in RL_versus_LL.

As shown in Figure 5, the metabolite Naringenin‐4'‐O‐glucoside appeared to be correlated with several crucial genes in the RL_versus_LL comparison group: *CsNEET* (CDGSH iron–sulfur domain‐containing protein NEET; *CsaV3_5G005490*), CsNAC6 (NAC domain‐containing protein 6; *CsaV3_6G012740*), *CsUBC25* (E2 ubiquitin‐conjugating enzyme 25; *CsaV3_6G028570*), and *CsGPT2* (glucose‐6‐phosphate/phosphate translocator 2; *CsaV3_6G049170*). *CsNEET*, which is expressed in chloroplasts and involved in “leaf senescence” (GO:0010150), and CsNAC6 were negatively correlated with Naringenin‐4'‐O‐glucoside levels in the RL_versus_LL comparison. On the other hand, *CsUBC25*, involved in the “protein K63‐linked ubiquitination” (GO:0070534) process, and *CsGPT2*, which regulates “photosynthesis” (GO:0010109), were positively correlated with the Naringenin‐4'‐O‐glucoside level.

Additionally, *CsDIRL1* (Protein Defective in Induced Resistance 1; *CsaV3_7G018790*) was positively correlated with levels of both L‐pipecolic acid and LysoPC 16:1 in the RL_versus_LL comparison. CsDIRL1 is involved in the “salicylic acid mediated signaling pathway” (GO:0009862) and “response to stress” (GO:0006950). Furthermore, LysoPC 16:1 content was positively correlated with *CsTRPB* (tryptophan synthase beta chain 2; *CsaV3_1G010980*), *CsCRK25* (cysteine‐rich receptor‐like protein kinase 25; *CsaV3_1G011180*), and *CsMYB3* (transcription factor MYB3; *CsaV3_3G043510*). The relationships between these DEGs and low‐temperature stress warrant further investigation to clarify their roles in cucumber's response to cold stress.

## 4 Discussion

Accumulating evidence indicates that plant hormones are of the essence for plants to accommodate to low‐temperature stresses (Chen et al. 2016). Auxin, a central component in many hormone regulatory networks, is especially significant in plant adaptability and developmental processes (Chen et al. 2016). Prior studies have highlighted auxin's involvement in leaf development and color changes in cucumber, as well as its fundamental role in flower formation (Chen et al. 2016; Sharif et al. 2022; Song et al. 2018; Zhang, Li, et al. 2020). Comparisons between resistant “Zhongnong37” and cold‐sensitive “Shuyanbailv” cultivars revealed higher levels of IAA, ABA, and JA in the resistant cultivars across three low‐temperature treatments (15°C–10°C, 12°C–8°C, and 9°C–5°C) (Amin et al. 2022). For instance, cucumber reduces the expression of GA20‐oxidase and GA3‐oxidase, thereby lowering the levels of active GA to inhibit root growth (Bai et al. 2016). Treatment with H_2_S led to a substantial increase in endogenous IAA, thereby enhancing cold tolerance, while application of 1‐naphthylphthalamic acid (NPA) attenuated the chilling tolerance induced by H_2_S through lowering the IAA level (Zhang, Liu, et al. 2020). In *Arabidopsis thaliana* , the root growth rate and gravitropic response decreased by 50% after 8–12 h of root zone cold stress at 4°C, due to cold stress inhibiting auxin transport mediated by PIN2 (Shibasaki et al. 2009). In our study, low‐temperature stress induced a significant increase of auxin proportions from 88% in RL, to 99% in SL and 94% in LL, showing the consistent results with previous studies. However, the ABA level in SL is dramatically decreased when compared to RL and partially restored in LL in this study, suggesting a dynamic regulation relationship between ABA and auxin contents in plants under different low‐temperature stresses.

Among the metabolites analyzed, flavanone, LPC, organic acid, and phenolic acids were notably more abundant in cucumber leaves, with flavanones being the most prevalent. Flavanones, a subgroup of flavonoids characterized by a saturated C‐ring, are crucial in enhancing plant cold tolerance, although most studies to date have focused on their role in freezing rather than chilling stress (Zhuang et al. 2023). In this study, flavanone levels in cucumber leaves rose up to 59% in response to chilling stress, highlighting their potential importance under low temperatures. Organic acids also play a role in low‐temperature resistance, with fumaric acid and azelaic acid contributing to cold tolerance in *Arabidopsis* (Dyson et al. 2016). Moreover, our results showed significantly higher L‐pipecolic acid in LL, suggesting that it may enhance low‐temperature tolerance in cucumber leaves. Additionally, phenolic acids functions in cold acclimation, as seen in rice, where more bound flavonoids, phenolics, and phenolic acids were found in cold tolerant rice (Rayee et al. 2020). In our study, phenolic compounds, particularly naringenin‐4'‐o‐glucoside to flavonoids, are essential for coping with cold acclimation in cucumber leaves, while *α*‐hydroxycinnamic acid is vital for adaptation of cucumber leaves to suboptimal temperature. Naringenin‐4'‐O‐glucoside, L‐pipecolic acid, and LysoPC 16:1 emerge as potential candidates for molecular breeding to enhance cold stress tolerance and promote terminal flower formation in cucumber.

The *CsFT* gene, as one member of the phosphatidy phosphatidylethanolamine‐binding protein (BEBP) family, promotes flower formation and inhibits plant flowering (Guo and Huang 2014). The four FT homologous genes in tobacco, *NtFT1*, *NtFT2*, *NtFT3*, and *NtFT4*, were found to express in leaves. Intriguingly, overexpression of *NtFT4* promotes tobacco flowering, while overexpression of *NtFT1*, *NtFT2*, and *NtFT3* inhibit it (Harig et al. 2012). In cucumber, *CsTFL1* and *CsFT* function antagonistically in regulating determinate growth and terminal flowering, with *CsFT* promoting flowering and *CsTFL* inhibiting it (Wen et al. 2019). In our study, temperature decrease led to a decline in *CsFT* expression, aligning with delayed determinate growth and terminal flower formation. This might be attributed to the upregulation of specific DETFs, such as *CsTI10A* and *CsNAC6*, which warrants further exploration. Intriguingly, a plethora of evidences have shown that TFs of the NAC family are involved in flowering regulation, such as GMNAC81, a positive regulator of plant flowering (Pimenta et al. 2016; Yoo et al. 2007). In contrast, NAC075 and NAC2 in *Picea wilsonii* suppress plant flowering (Fujiwara and Mitsuda 2016; Zhang et al. 2018). The role of FT in flower formation remains critical, and additional research into its regulatory pathways in cucumber is needed.

Plant cold response signaling is typically categorized into CBF‐dependent and CBF‐independent pathways. The ICE‐CBF‐COR pathway is an analytic pathway, where ICE proteins upregulate CBF, which subsequently activates COR genes through promoter binding (Liu et al. 2018; Ramezani et al. 2017; Tang et al. 2022). However, recent studies suggest that TFs in *AP2/ERF* and *WRKY* families regulate plant hypothermia responses through CBF‐independent pathways. In recent years, there has been some progress in understanding the molecular mechanisms of cucumber's response to low‐temperature stress. For example, cucumber cells respond to low‐temperature stress by upregulating the expression of downstream *CsICE* and *CsCBF* core cold tolerance genes through the CsGPA1‐CsCOR413PM2‐Ca^2+^ signaling pathway (Tang et al. 2022). In terms of cold acclimation, the high expression of the heat shock protein gene *CsHSFA1d* can enhance cucumber's adaptability to low temperatures. The mechanism is that *CsHSFA1d* increases the JA content in cucumber seedlings, leading to the degradation of CsJAZ5, thereby releasing CsICE1 to activate the ICE‐CBF‐COR signaling pathway, ultimately improving cold acclimation (Qi et al. 2022). Additionally, exogenous application of N‐acetyl‐5‐methoxytryptamine can significantly enhance the transcription of *Nitrate Reductase* (NR), protein activity, and accumulation of NO, thereby enhancing cucumber's adaptation to low temperatures by activating the antioxidant system and the ICE1‐CBF1‐COR47 signaling pathway (Feng et al. 2021). Regarding suboptimal temperature response in cucumber root zones, suboptimal temperatures reduce nitrogen absorption in the roots by lowering GA levels, while exogenous application of GA can increase nitrogen accumulation in the roots (Bai et al. 2016). At the plant physiology level, low temperatures can induce the accumulation of plant hormones such as auxin and JA in the roots, thereby improving cucumber's adaptation to low temperatures (Sun et al. 2024). In this study, *CsCBF* did not show differential expression across the three samples, whereas *AP2* family DETFs were significantly upregulated by chilling stress. The findings imply that cucumber leaves may rely on the AP2/ERF family rather than CBF for cold response regulation. Nonetheless, further research is essential to confirm this hypothesis.

## 5 Conclusion

This study provides a comprehensive profile of phytohormones, DEMs, and DEGs in cucumber leaves exposed to room temperature, suboptimal, and low‐temperature conditions. Our findings highlight auxin as the most abundant phytohormone under these conditions, accounting for 99% of total hormones in SL and 94% in LL treatments. In contrast, the levels of ABA, JA, and ST significantly declined in both SL and LL treatments. This dominance of auxin, particularly in SL and LL, underscores its crucial role in leaf development, chilling injury response, and terminal flower formation. In metabolite profiling, flavanones emerged as the most abundant group, comprising over half of the total metabolites in cucumber leaves. Notably, flavanone accumulation increased with chilling stress, with Naringenin‐4'‐O‐glucoside (HJN087) identified as the primary flavanone. The proportions of LPC and organic acids remained stable across temperature treatments, whereas phenolic acid levels dropped significantly from 13% at room temperature to 4% under chilling stress. The RNA‐seq analysis identified 94 DEGs between room temperature and low‐temperature (RL_vs._LL) conditions, 20 DEGs between suboptimal and low temperature (SL_vs._LL), and 5 DEGs between room and suboptimal temperatures (RL_vs._SL). Notably, no DEGs were common across all three comparisons, suggesting distinct mechanisms of cold acclimation as plants transition from room temperature to suboptimal and chilling conditions. Co‐enrichment analysis illustrated that DEGs and DEMs were significantly enriched in secondary metabolite biosynthesis, cysteine and methionine metabolism (ko00270), glycerophospholipid metabolism (ko00564), and phenylpropanoid biosynthesis (ko00940) in RL_versus_LL. In SL_versus_LL, DEGs and DEMs were notably co‐enriched in metabolic pathways, cofactor biosynthesis (ko01110), and secondary metabolite biosynthesis. In terms of DETFs, CsaV3_3G012170 (*AP2/ERF*), CsaV3_3G006120 (*NAC*), and CsaV3_1G041270 (*TIFY*) were upregulated in both SL and LL, suggesting their potential positive regulation of cucumber chilling tolerance. Conversely, *CsaV3_1G044210* (*FT*), *CsaV3_6G012740* (*NAC*), and *CsaV3_3G026410* (*HB/HD‐ZIP*) were transcriptional suppressed in both SL and LL. In summary, our study elucidates the distinct phytohormone, gene expression, and metabolite profiles that cucumber leaves exhibit in response to chilling stresses, advancing our perception on the molecular mechanisms underlying cucumber cold tolerance.

## Author Contributions

**Shijun Sun:** formal analysis (equal), funding acquisition (equal), investigation (equal), writing – original draft (equal). **Shuiyuan Hao:** funding acquisition (equal). **Ye Liu:** formal analysis (equal). **Xiaoni Gao:** investigation (equal). **Tianlei Mu:** methodology (equal). **Xu Zhang:** investigation (equal). **Yusong Luo:** data curation (equal), writing – original draft (equal), writing – review and editing (equal). **Zhengnan Li:** supervision (equal), writing – review and editing (equal).

## Ethics Statement

It is not applicable for humans or animals. The work is implemented in plants, not in human beings or animals.

## Conflicts of Interest

The authors declare no conflicts of interest.

## Supporting information


Figure S1

Figure S2

Figure S3

Tables S1–S5

## Data Availability

The authors confirmed that the data supporting the findings of this study are available within the article and its Supporting Information.
