# Supplementary material for: Integrative Profiling of Phytohormones, Metabolomics, and Transcriptomics Reveals Key Regulators of Cold Tolerance in Cucumber Leaves
Source: Food Sci Nutr. 2025 Mar 2;13(3):e70027. doi: 10.1002/fsn3.70027 (PMC11873373; doi:10.1002/fsn3.70027)
Supplement: Supplementary file 1 — Figure S1 [file FSN3-13-e70027-s002.docx]

**
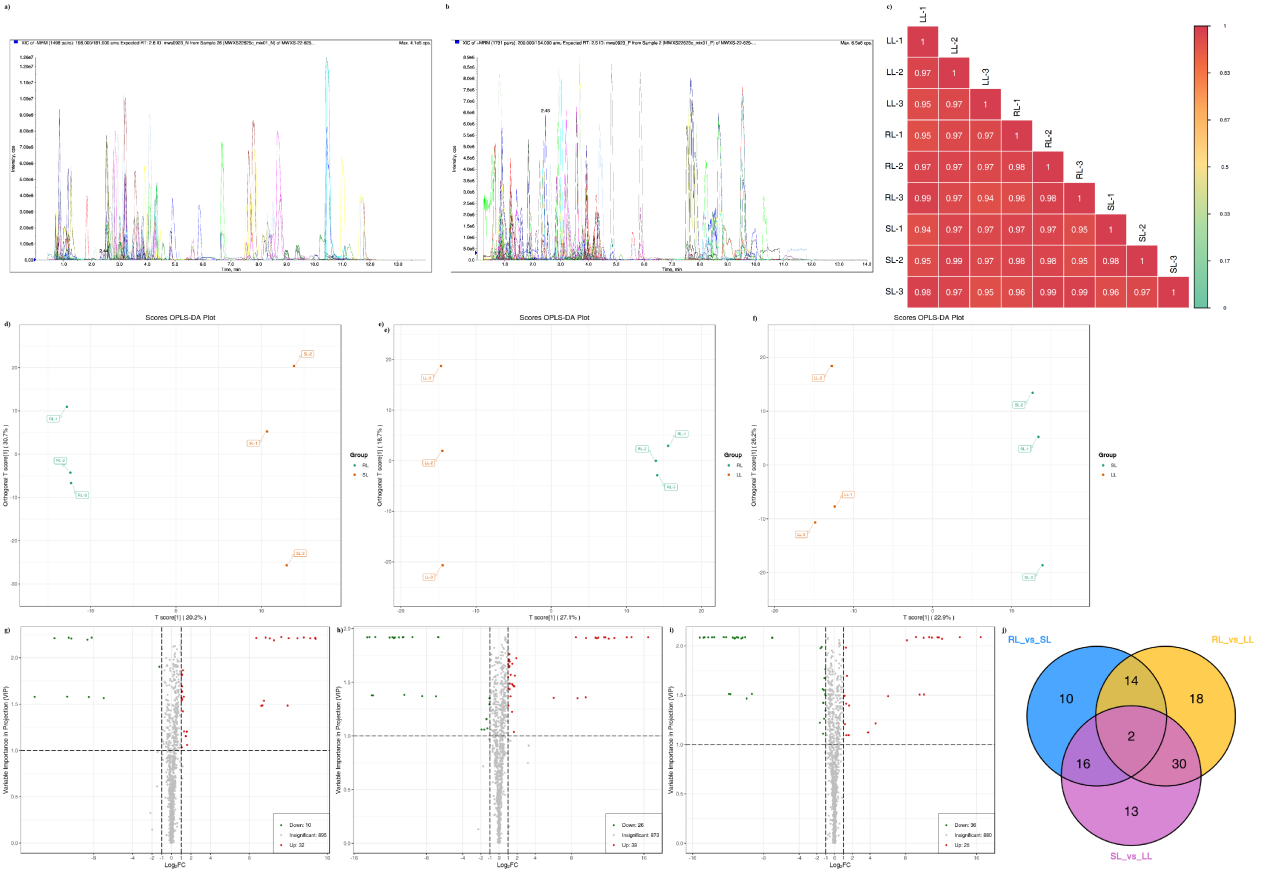
**

**Supplementary Figure 1 MRM detection of multimodal maps-N (a) and P (b) of cucumber leaf samples. Correlation analysis (c) between repeat leaf samples of cucumber. Orthogonal partial Least Squares-Discriminant analysis of RL_vs_SL (d), RL_vs_LL (e) and SL_vs_LL (f). DAM Volcano Map of RL_vs_SL (g), RL_vs_LL (h) and SL_vs_LL (i). Venn diagram (j) depicting the shared and specific metabolites in the RL, SL and LL. RL: The leaves of cucumber in room temperature condition; SL: The leaves of cucumber in suboptimal temperature condition; LL: The leaves of cucumber in low temperature condition.**
