# Supplementary material for: Integrative Profiling of Phytohormones, Metabolomics, and Transcriptomics Reveals Key Regulators of Cold Tolerance in Cucumber Leaves
Source: Food Sci Nutr. 2025 Mar 2;13(3):e70027. doi: 10.1002/fsn3.70027 (PMC11873373; doi:10.1002/fsn3.70027)
Supplement: Supplementary file 2 — Figure S2 [file FSN3-13-e70027-s003.docx]

**
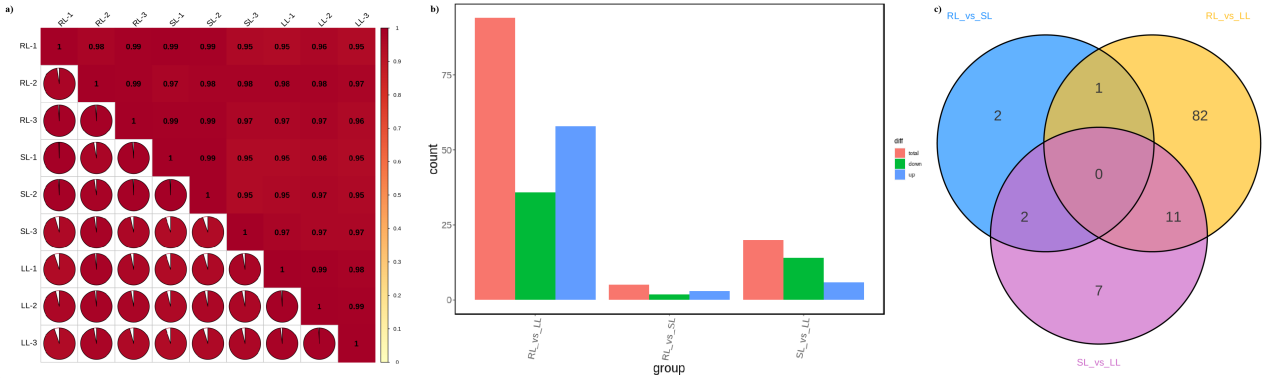
**

**Supplementary Figure 2 Correlation (a) between RNA-seq repeat leaf samples of cucumber. The statistics of differential genes in leaf of cucumber. b): The statistics of differential genes in leaf, the vertical axis represents the number of differential genes; Bar values are average on three replicates. Venn diagram (c) depicting the shared and specific genes in the RL, SL and LL. RL: The leaf of cucumber in room temperature condition; SL: The leaf of cucumber in suboptimal temperature condition; LL: The leaf of cucumber in low temperature condition.**
