# Supplementary figures and images for: Integrative Profiling of Phytohormones, Metabolomics, and Transcriptomics Reveals Key Regulators of Cold Tolerance in Cucumber Leaves
Source: Food Sci Nutr. 2025 Mar 2;13(3):e70027. doi: 10.1002/fsn3.70027 (PMC11873373; doi:10.1002/fsn3.70027)

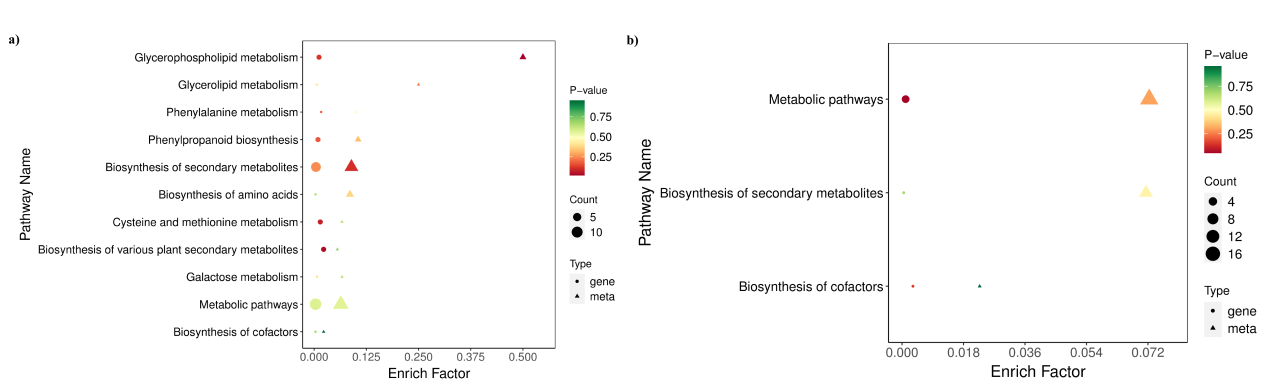
**Supplementary figure 3. The KEGG enrichment of DEGs and DAMs in RL_vs_LL (j) and SL_vs_LL (k).**

Supplement: Supplementary file 3 — Figure S3 [file FSN3-13-e70027-s001.docx]
